# Markerless Measurement and Evaluation of General Movements in Infants

**DOI:** 10.1038/s41598-020-57580-z

**Published:** 2020-01-29

**Authors:** Toshio Tsuji, Shota Nakashima, Hideaki Hayashi, Zu Soh, Akira Furui, Taro Shibanoki, Keisuke Shima, Koji Shimatani

**Affiliations:** 1 Department of System Cybernetics, Graduate School of Engineering, Hiroshima University, 1-4-1 Kagamiyama, Higashi-Hiroshima, Hiroshima, 739-8527 Japan; 2 Faculty of Information Science and Electrical Engineering, Kyushu University, 744 Motooka, Nishi-Ku, Fukuoka, Fukuoka, 819-0395 Japan; 3 Faculty of Engineering, Ibaraki University, 4-12-1 Nakanarusawa, Hitachi, Ibaraki, 316-8511 Japan; 4 Faculty of Engineering, Yokohama National University, 79-1 Tokiwadai, Hodogaya Ward, Yokohama, Kanagawa 240-8501 Japan; 5 Department of Physical Therapy, Prefectural University of Hiroshima, 1-1 Gakuen, Mihara, Hiroshima, 723-0053 Japan

**Keywords:** Movement disorders, Neurodevelopmental disorders, Biomedical engineering

## Abstract

General movements (GMs), a type of spontaneous movement, have been used for the early diagnosis of infant disorders. In clinical practice, GMs are visually assessed by qualified licensees; however, this presents a difficulty in terms of quantitative evaluation. Various measurement systems for the quantitative evaluation of GMs track target markers attached to infants; however, these markers may disturb infants’ spontaneous movements. This paper proposes a markerless movement measurement and evaluation system for GMs in infants. The proposed system calculates 25 indices related to GMs, including the magnitude and rhythm of movements, by video analysis, that is, by calculating background subtractions and frame differences. Movement classification is performed based on the clinical definition of GMs by using an artificial neural network with a stochastic structure. This supports the assessment of GMs and early diagnoses of disabilities in infants. In a series of experiments, the proposed system is applied to movement evaluation and classification in full-term infants and low-birth-weight infants. The experimental results confirm that the average agreement between four GMs classified by the proposed system and those identified by a licensee reaches up to 83.1 ± 1.84%. In addition, the classification accuracy of normal and abnormal movements reaches 90.2 ± 0.94%.

## Introduction

UNICEF reported in “The State of the World’s Children 2016” that approximately 10% of newborn babies in Japan have a low birth weight (LBW), and this percentage is expected to increase in the near future^1^. Although early interventions such as stand training, gait training, and cognitive training are efficient approaches for easing the disabilities that may result from LBW^2^, a reliable technique for early diagnosis is required.

Previous studies have identified a relationship between motor abnormality and various disabilities^3^, and methods for early diagnosis have focused on the characteristics of motor function. For example, the Brazelton neonatal assessment^4^ evaluates infant motor responses to auditory and optical stimulation. The Vojta method^5^ encourages infants to take certain postures to evoke a motor response and evaluates the correspondence with developmental stages. However, these methods require infants to be restrained for long periods and involve the application of stimuli that can be a mental and physical burden.

In contrast to the above approaches, the Prechtl’s method for the assessment of general movements (GMs)^6^ has attracted attention because it allows motor-function-based diagnosis without the need to apply stimuli. GMs are part of spontaneous movements in an infant that are associated with neural development, and they can be classified into two types of normal movement and five types of abnormal movement^7^ (Table 1). GMs can only be assessed by well-trained clinicians who have an assessment license^8^. In addition, as this approach requires the clinician to observe GMs over a long period of time, it places a considerable burden on licensees and makes it difficult to perform objective and quantitative evaluations.

**Table 1:** Taxonomy of GMs.

Corrected age (weeks)	Normal GMs	Abnormal GMs
0–9	Writhing movements (WMs)	Poor repertoire of GMs (PR)
		Cramped-synchronized GMs (CS)
		Chaotic GMs (Ch)
6–20	Fidgety movements (FMs)	Absent fidgety movements (aFMs)
		Abnormal fidgety movements (abnFMs)

Therefore, we describe herein a markerless movement measurement and evaluation system for GMs. We used video analysis and machine learning to classify infants’ movements based on Prechtl’s GM assessment from video images during GMs. The proposed system uses a video camera to measure infants’ movements without attaching markers to their bodies, and it extracts evaluation indices reflecting various aspects of these movements to enable objective evaluation. The movement types, based on the clinical definition of GMs, are automatically classified using a neural network with a stochastic structure, thereby supporting an early diagnosis by detecting motor abnormalities.

## General Movements and Their Evaluation

### General movements

GMs are typical spontaneous whole-body movements that are most frequently observed in infants, and they last from several seconds to several minutes. These movements start to appear 8–9 weeks after fertilization, and they disappear after 15–20 weeks, along with the appearance of voluntary movements. Prechtl stated that GMs could be generated by the central pattern generator embedded from the brain stem to the spinal cord, and the features of these movements may alter with the development of the brain cortex^3^. Current research suggests that GMs reflect neuronal development; thus, a method has been established to evaluate neuronal development through the observation of GMs^6^. In addition, the evaluation of GMs has been reported to have either equal or better efficacy for prognostic predictions as neurological tests^9^, and it is therefore beneficial in the diagnosis of future disorders.

Based on their characteristics, GMs can be classified into two types of normal movement and five types of abnormal movement, as described in Table 1. For example, normal GMs are classified as writhing movements (WMs) or fidgety movements (FMs). WMs appear until weeks 6–9 and are characterized by the limbs tracing an ellipse and the extension of the upper limbs. FMs appear as an alternative to WMs from weeks 6–9 weeks until weeks 15–20, and they are characterized by kicking movements. Poor repertoire GMs (PR) are abnormal movements that can appear until weeks 6–9 and consist of monotonic movements that lack variety. A PR pattern seems to be associated with minor neurological dysfunctions^10^. Cramped-synchronized GMs (CS) can appear in the same period, and they are characterized by stiff movements and lack of fluidity^7^. It has been reported that observation of CS is highly predictive of the development of cerebral palsy^11,12^.

### Objective evaluation of infant movements

To enable the objective evaluation and measurement of infant movements, the early detection of disorders in infants has been extensively studied. For example, Algra *et al*. measured myoelectric potentials by attaching electrodes to infants’ limbs and evaluating the muscular activity and duration of movement during normal GMs^13,14^. Several studies have used position sensors and acceleration sensors to evaluate the spontaneous movement of limbs during GMs^15–20^. Specifically, Heinze *et al*.^18^ used acceleration sensors attached to the four limbs to analyse the periodicity of velocity and acceleration in spontaneous movements, and they reported that the extracted features can be used to diagnose movement disorders. However, the above studies required sensors or markers to be attached to the infants, which can disturb the spontaneous emergence of their movements.

As an alternative, markerless measurement methods for infant movements have also been developed^21–23^. For example, our group proposed a video analysis method to analyse infant movements and enable objective evaluation^21^. Adde *et al*. extracted several features including the magnitude of whole-body movements from video images to classify two types of GMs^22^. This study successfully detected two types of GMs and enabled prognosis predictions of cerebral palsy.

As described above, previous studies have demonstrated the effectiveness of objective and automatic GM evaluation in clinical practice. However, the evaluable movements represent only a portion of all GMs. To classify various GMs, it is necessary to analyse the movements of different regions of the body from various aspects. The next section describes the proposed system, which enables such analysis.

## Proposed System

**Figure 1:**
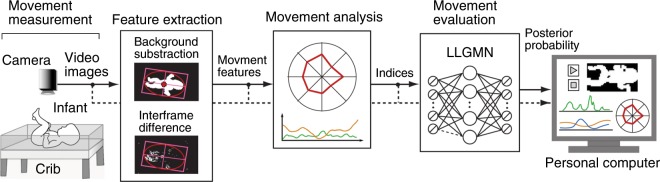
Overview of the proposed GM evaluation system.

Figure 1 shows the proposed markerless movement measurement and evaluation system for GMs. The proposed system consists of five parts: movement measurement, feature extraction, movement analysis, GM-based movement classification, and output/storage of results. This section describes the configuration of the proposed system.

### Movement measurement module

The movement of infants is measured using a video camera fixed directly above and parallel to the crib surface, which is covered by a unicolor fabric spread, as shown in the left-hand side of Fig. 1. The infant is assumed to be lying supine at the centre of the crib, and the height of the video camera is adjusted so that the whole of the infant’s body can be captured within the video frame. The video images are recorded and stored on a personal computer with a frame rate of *f*_s_ Hz.

### Feature extraction

**Figure 2:**
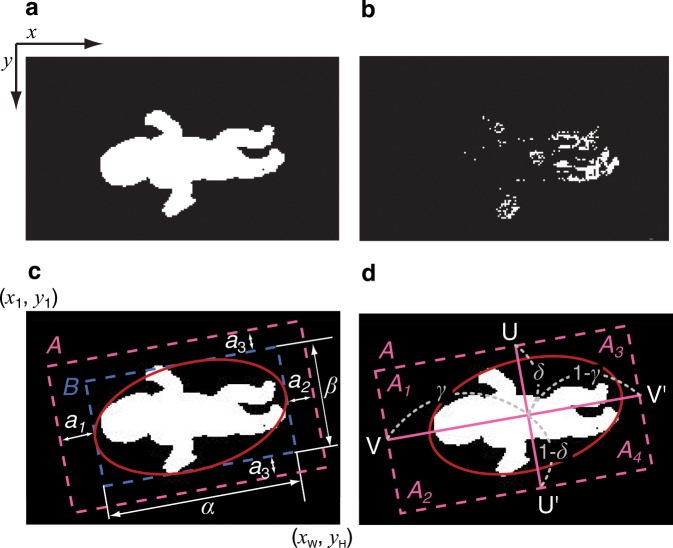
Feature-extracted images. (**a**) Background difference image. (**b**) Interframe difference image. (**c,d**) Procedure of the image segmentation. (**c)** Approximated ellipse and analysis area. (**d**) Line segments and divided areas.

The feature extraction follows the method adopted by Shima *et al*.^21^. Figure 2 shows the feature extraction images. The coordination of the image is defined as follows: the origin is defined as the top-left pixel, *W* represents the maximum pixel number along the *x* axis, and *H* represents the maximum pixel number along the *y* axis. That is, the *x* axis is defined in the range *x*_*w*_ ($w=1,2,\,\cdots ,\,W$) and the *y* axis, in the range *y*_*h*_ ($h=1,2,\,\cdots ,\,H$). The proposed system converts the measured images to grayscale images, and it calculates the background subtraction to generate binary images using a brightness threshold *T*, where black (0) represents the background area and white (1) represents the infant’s body (Fig. 2a). At the same time, interframe difference images are calculated between time-adjacent frames to generate additional binary images using a brightness threshold *T*, where white (1) represents a pixel in which infant movement has been detected (Fig. 2b). To extract the movement of all four limbs, the whole-body area is determined and divided into four areas using two line segments, as shown in Fig. 2c,d. This process uses the following algorithm. First, ellipse approximation based on the least-squares method is applied to the background difference image to generate an ellipse surrounding the outline of the body. This allows the area *B* representing the circumscribed rectangle of the ellipse to be determined (Fig. 2c). By letting *α* and *β* represent the pixel numbers of the long and short axes of the rectangle, respectively, the analysis area *A* is determined as shown in Fig. 2c by adding the margins ${a}_{1}={t}_{{a}_{1}}\alpha$ pixels and ${a}_{2}={t}_{{a}_{2}}\alpha$ pixels to the ends of the long axis and ${a}_{3}={t}_{{a}_{3}}\beta$ pixels to both ends of the short axis, where ${t}_{{a}_{1}}$, ${t}_{{a}_{2}}$, and ${t}_{{a}_{3}}$ are adjustable variables. The analysis area *A* is then divided into four areas by two line segments as shown in Fig. 2d: the line segment UU′ divides *A* into the upper and lower body areas with ratios of $\gamma \mathrm{:(1}-\gamma )$, and the line segment VV′ divides *A* into the left and right body areas with ratios of $\delta \mathrm{:(1}-\delta )$. The divided areas are represented by the variables *A*_*k*_ ($k\,=\,\mathrm{1,}\,\cdots \,\mathrm{,}\,4$), where *A*_1_ represents the left upper body; *A*_2_, the right upper body; *A*_3_, the left lower body; and *A*_4_, the right lower body. The combination of these four areas generates *A*_*k*_ ($k\,=\,\mathrm{5,}\,...\mathrm{,}\,9$), where *A*_5_ represents the upper body; *A*_6_, the lower body; *A*_7_, the left body; *A*_8_, the right body; and *A*_9_, the whole body.

To extract features from infant movements, the changes in body posture ${}^{({A}_{k})}\,{P}_{l}$ and those in body movement ${}^{({A}_{k})}\,{M}_{l}$ are extracted from each analysis area *A*_*k*_ ($k\,=\,\mathrm{1,}\,...\mathrm{,}\,9$). The velocity of the body centre *G*^v^ (${G}_{l,x}^{{\rm{v}}}$, ${G}_{l,y}^{{\rm{v}}}$) and the fluctuation of the body centre *G*^d^ in a frame window (${G}_{l,x}^{{\rm{d}}}$, ${G}_{l,y}^{{\rm{d}}}$) are also calculated from the area *A*_9_. Here, *l* ($l=\mathrm{1,}\,\mathrm{2,}\,...,L$) represents the frame number of the video image, and *L* is the total number of frames in each analysis interval.

A change in body posture ${}^{({A}_{k})}\,{P}_{l}$ is defined by a change in the whole body area, and a change in movement ${}^{({A}_{k})}\,{M}_{l}$ is defined as the magnitude of movement normalized by the whole body area using the following equations:

1 $${}^{({A}_{k})}\,{P}_{l}=\mathop{\sum }\limits_{w\mathrm{=1}}^{W}\,\mathop{\sum }\limits_{h\mathrm{=1}}^{H}\,{}^{({A}_{k})}\,{O}_{l}({x}_{w},{y}_{h}),$$

2 $$\begin{array}{rcl}{}^{({A}_{k})}\,{M}_{l} & = & \frac{\mathop{\sum }\limits_{w\mathrm{=1}}^{W}\,\mathop{\sum }\limits_{h\mathrm{=1}}^{H}\,{}^{({A}_{k})}\,{O^{\prime} }_{l}({x}_{w},{y}_{h})}{{}^{({A}_{9})}\,{P}^{{\rm{ave}}.}},\\ {}^{({A}_{k})}\,{O^{\prime} }_{l}({x}_{w},{y}_{h}) & = & |{}^{({A}_{k})}\,{O}_{l}({x}_{w},{y}_{h})-{}^{({A}_{9})}\,{O}_{l-1}({x}_{w},{y}_{h})|,\end{array}$$

where ${}^{({A}_{k})}\,{O}_{l}({x}_{w},{y}_{h})$ represents the binary number of the pixel at coordinates $({x}_{w},{y}_{h})$ of the background difference image within analysis area *A*_*k*_. Note that ${}^{({A}_{k})}\,{O^{\prime} }_{1}({x}_{w},{y}_{h})=0$; ${}^{({A}_{9})}\,{P}^{{\rm{ave}}.}$ is the average of the maximum value of ${}^{({A}_{k})}\,{P}_{l}$ up to the *E*-th ($E\le L$) frame. The velocity of the body centre (${G}_{l,x}^{{\rm{v}}}$, ${G}_{l,y}^{{\rm{v}}}$) is defined as the difference in body centre coordinates between time-adjacent frames, and the fluctuation of the body centre (${G}_{l,x}^{{\rm{d}}}$, ${G}_{l,y}^{{\rm{d}}}$) is calculated from the average body centre coordinates (${G}_{x}^{{\rm{ave}}.}$, ${G}_{y}^{{\rm{ave}}.}$) over *L* frames using the following equations:

3 $$\left({G}_{l,x}^{{\rm{v}}},{G}_{l,x}^{{\rm{v}}}\right)=\left(\frac{{G}_{l,x}-{G}_{l-\mathrm{1,}x}}{\sqrt{{}^{\left({A}_{9}\right)}\,{P}^{{\rm{ave}}.}}},\frac{{G}_{l,y}-{G}_{l-\mathrm{1,}y}}{\sqrt{{}^{\left({A}_{9}\right)}\,{P}^{{\rm{ave}}.}}}\right),$$

4 $$\left({G}_{l,x}^{{\rm{d}}},{G}_{l,y}^{{\rm{d}}}\right)=\left(\frac{{G}_{l,x}-{G}_{x}^{{\rm{ave}}.}}{\sqrt{{}^{\left({A}_{9}\right)}\,{P}^{{\rm{ave}}.}}},\frac{{G}_{l,y}-{G}_{y}^{{\rm{ave}}.}}{\sqrt{{}^{\left({A}_{9}\right)}\,{P}^{{\rm{ave}}.}}}\right),$$

where the body centre coordinates *G*_*l,x*_, *G*_*l,y*_ are calculated from the background difference image using the following equations:

5 $${G}_{l,x}=\frac{1}{{}^{({A}_{9})}\,{P}_{l}}\mathop{\sum }\limits_{w\mathrm{=1}}^{W}\,\mathop{\sum }\limits_{h\mathrm{=1}}^{H}\,{x}_{w}\,{}^{({A}_{9})}\,{O}_{l}({x}_{w},{y}_{h}),$$

6 $${G}_{l,y}=\frac{1}{{}^{({A}_{9})}\,{P}_{l}}\mathop{\sum }\limits_{w\mathrm{=1}}^{W}\,\mathop{\sum }\limits_{h\mathrm{=1}}^{H}\,{y}_{h}\,{}^{({A}_{9})}\,{O}_{l}({x}_{w},{y}_{h}),$$

where $${G}_{\mathrm{1,}x}^{{\rm{v}}}=0$$ and $${G}_{\mathrm{1,}y}^{{\rm{v}}}=0$$.

### Movement analysis

By using the extracted features, movement analysis is performed based on clinical insights to calculate objective evaluation indices. Specifically, the proposed system generates *J* indices (Table 2) from four different aspects: (I) movement magnitude, including duration and magnitude of the movement; (II) movement balance, described by the ratio and correlation of movements between analysis areas; (III) movement rhythm, representing the periodicity of movement; and (IV) movement of the body centre. The details of calculation for each index are presented in the Appendix.

**Table 2:** Description of the evaluation indices.

Category	Index	Description
(I) Movement magnitude	${}^{({A}_{k})}\,{I}_{1}$	Movements frequency
	${}^{({A}_{k})}\,{I}_{2}$	Movements strength
	${}^{({A}_{k})}\,{I}_{3}$	Movements count
(II) Movement balance	${}^{({k}_{1},{k}_{2})}\,{I}_{4}$	Ratio of index ${}^{({A}_{k})}\,{I}_{1}$ in upper-limb and lower-limb
	${}^{({k}_{1},{k}_{2})}\,{I}_{5}$	Ratio of index ${}^{({A}_{k})}\,{I}_{2}$ in upper-limb and lower-limb
	${}^{({k}_{1},{k}_{2})}\,{I}_{6}$	Symmetry in upper-limb and lower-limb
(III) Movement rhythm	${}^{({A}_{k})}\,{I}_{7}$	Rhythm of ${}^{({A}_{k})}\,{M}_{l}$
	${}^{({A}_{k})}\,{I}_{8}$	Standard deviation of index ${}^{({A}_{k})}\,{I}_{7}$
	${}^{({A}_{9})}\,{I}_{{9}_{x}}$, ${}^{({A}_{9})}\,{I}_{{9}_{y}}$	Rhythm of *G*^v^
	${}^{({A}_{9})}\,{I}_{{10}_{x}}$, ${}^{({A}_{9})}\,{I}_{{10}_{y}}$	Standard deviation of index ${}^{({A}_{9})}\,{I}_{{9}_{x}}$, ${}^{({A}_{9})}\,{I}_{{9}_{y}}$
	${}^{({A}_{9})}\,{I}_{{11}_{x}}$, ${}^{({A}_{9})}\,{I}_{{11}_{y}}$	Rhythm of *G*^d^
	${}^{({A}_{9})}\,{I}_{{12}_{x}}$, ${}^{({A}_{9})}\,{I}_{{12}_{y}}$	Standard deviation of index ${}^{({A}_{9})}\,{I}_{{11}_{x}}$, ${}^{({A}_{9})}\,{I}_{{11}_{y}}$
(IV) Movement of the body centre	${}^{({A}_{9})}\,{I}_{{13}_{x}}$, ${}^{({A}_{9})}\,{I}_{{13}_{y}}$	Total magnitude of *G*^v^
	${}^{({A}_{9})}\,{I}_{{14}_{x}}$, ${}^{({A}_{9})}\,{I}_{{14}_{y}}$	Total magnitude of *G*^d^

### Motion classification based on GMs

First, each index is reduced to its canonical form based on the average *μ*_*j*_ (*j* = 1, 2, …, *J*) and the standard deviation *σ*_*j*_ of the corresponding index collected from the standard subject group, whose motions were clinically preclassified as the normal GMs.

7 $${z}_{j}=\frac{({I}_{j}-{\mu }_{j})}{{\sigma }_{j}},$$

where *z*_*j*_ represents each canonicalized index. As a result of canonicalization, the indices of the target motion can be evaluated in terms of the variation from the indices of the standard group, as the indices are expected to follow the normal distribution ${\mathscr{N}}\mathrm{(0,}\,\mathrm{1)}$.

A feedforward-type neural network called the log-linearized Gaussian mixture network (LLGMN)^24^ is used as the classifier for the movements. LLGMN includes Gaussian mixture models in a log-linearized form, enabling the estimation of the probabilistic distribution of a given sample dataset. First, the parameters of the LLGMN are adjusted using learning samples of the *C* classes of GMs $\,\,z{}^{(n)}=[{z}_{1}^{(n)},\,{z}_{2}^{(n)},\,\cdots ,\,{z}_{J}^{(n)}{]}^{{\rm{T}}}\in \,\,{{\mathbb{R}}}^{J}$ ($n\,=\,1,\,2,\,...,\,{N}_{c}:{N}_{c}$ is the number of learning samples for each GM). After this learning process, the posterior probabilities $\,Y={[{Y}_{1},{Y}_{2},...,{Y}_{C}]}^{{\rm{T}}}$ corresponding to each class can be obtained by inputting a new set of canonicalized indices *z* obtained from the motion of the infant. Finally, the posterior probabilities calculated for every analysis interval (*L* frames) are averaged over the entire analysis interval, and the movement type of the infant is classified as the GM class with the highest posterior probability. This method enables the automatic classification of motion types corresponding to the predetermined *C* types of GM. In addition, to avoid misclassification caused by ambiguous input, the entropy *S* is employed. This is defined as

8 $$S=-\,\mathop{\sum }\limits_{c\mathrm{=1}}^{C}\,{Y}_{c}\,\log \,{Y}_{c}.$$

If *S* exceeds the threshold value *S*_th_, the input motion is classified as Type 0 (i.e., does not belong to any GM motion types). When the movement frequency of the upper or lower body is equal to 0, indicating no movement was detected, it is also classified as Type 0.

### Display and storage

**Figure 3:**
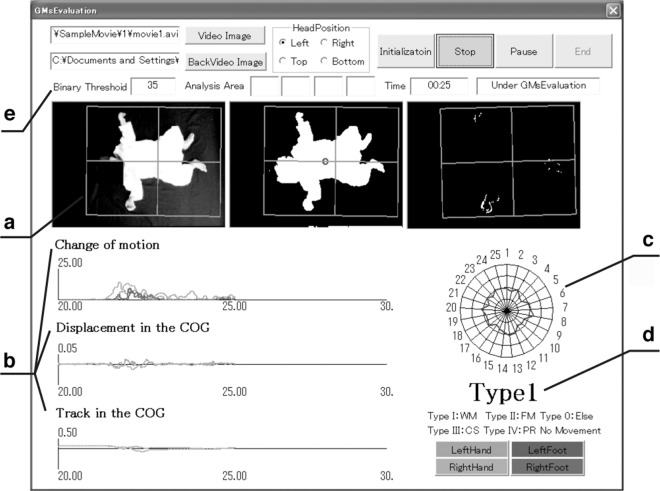
Screenshot of the proposed system. (**a**) Measured image, background difference image, and interframe difference image. (**b**) Time series waveforms of the changes in movement, velocity of the body centre, and fluctuation of the body centre. (**c**) Radar chart of the calculated indices. (**d**) Classification result based on GMs. (**e**) Users can set the threshold value *T* for binarization and the head position of the infant.

Figure 3 shows the display of the proposed system. Part (a) shows the measured image, background difference image, and interframe difference image. Part (b) shows the time series of changes in movement ${}^{({A}_{k})}\,{M}_{l}$, velocity of the body centre (${G}_{l,x}^{{\rm{v}}}$, ${G}_{l,y}^{{\rm{v}}}$), and fluctuation of the body centre (${G}_{l,x}^{{\rm{d}}}$, ${G}_{l,y}^{{\rm{d}}}$). Part (c) shows the calculated indices in the form of a radar chart, and (d) shows the classification results based on GMs. The user interface (e) allows users to set the threshold value *T* for binarization and the head position of the infant. This permits the analysis and classification configuration to be adjusted according to the video image quality. The display system also allows users to visually capture movement features and reconfirm the movements by rewinding the video image and analysis results in the event of abnormalities.

## Experimental Configuration

Movement analysis and GM classification experiments were conducted using the proposed system. The experiments were in full compliance with the Declaration of Helsinki, and they were performed under the approval of the Research Ethics Committee of the Prefectural University of Hiroshima and the ethics committee of the National Cerebral and Cardiovascular Center. Informed consent was obtained from the parents of the subjects, and the experimental purpose and methods were fully explained. Informed consent for publication of identifying information/images in an online open-access publication was also obtained from the parents of the subjects whose images appear in this manuscript.

Nineteen infants, including those with LBW, were studied. Subjects A–C were full-term infants, and subjects D–S were LBW infants. The video images were taken under doctor supervision to minimize any burden on the infants and avoid potential dangers. Video images of two additional subjects (T and U) were obtained from a DVD, “Spontaneous Motor Activity as a Diagnostic Tool,” by Prechtl^25^. Therefore, video images of 21 subjects were used in the experiments. The detailed information of the subjects is shown in Supplementary Table S1. The recording and analysis followed the clinical standards of Prechtl’s GM assessment, and periods of crying and sleeping were excluded from the analysis. The average video length was 442.17 ± 284.15 s per subject. The measurement and analysis parameters were set as follows: *f*_s_ = 30 Hz, ${t}_{{a}_{1}}\mathrm{=0.02}$, ${t}_{{a}_{2}}\mathrm{=0.02}$, ${t}_{{a}_{3}}\mathrm{=0.03}$, *W* = 720 pixels, *H* = 480 pixels, *E* = 10, *M*_th_ = 0.05, *f*_max_ = 5 Hz, *L* = 900 frames, and *L*_f_ = 300 frames. As the videos were taken under different situations, the ratio parameters for area division *γ* and *δ* were manually adjusted to ensure that the motion of infants was within the analysis area, and the threshold parameter *T* for binarization was manually adjusted to enable appropriate background difference images to be generated.

A second-order Butterworth filter was applied to smooth the changes in motion and velocity of the body centre, thus eliminating noise from the video images. The cut-off frequency of the filter applied to the change in motion was ${f}_{{\rm{cut}}}^{{\rm{m}}}=10$ Hz, and that to the change in body centre was ${f}_{{\rm{cut}}}^{{\rm{g}}}=5$ Hz. The fast Fourier transform was applied under a window width of 128 data points and an overlap of 127 data points. The normalization window width for the cross-correlation function (see Appendix) was 300 data points, and the overlap was set to 299 data points. The analysis focused on the upper body and lower body (*k* = 5, 6) and generated *J* = 25 indices. The indices were calculated every *S* frames using data in each analysis interval of length *L*. For subject A–S, we set *S* = *L*, meaning the analysis interval was not overlapped. For subject T and U, we set *S* = 1, meaning the analysis interval was overlapped by *L* − 1 frames. This is because their videos were considerably shorter than those of the other subjects.

In the experiment, motion images were first classified into four groups by physical therapists (PTs) licensed for GM evaluation. The PTs assessed GMs from the video recordings. This classification task was performed for every 30 s (900 frames) of video, and the classification result of each interval was determined as the most frequently appearing type of GM. In accordance with the official GM assessment guideline, the classification was conducted by a single PT; videos that were difficult to judge were assessed by multiple PTs. Analysis intervals where GMs can be clearly seen were also selected by PTs, and the assessment of GMs was conducted on these intervals. The number of times that each type of GM was classified was as follows: WMs: 193; FMs: 279; CS: 31; and PR: 66. Other types of GMs (e.g. abnormal FMs) were not observed in the video used in the experiments.

The proposed system automatically classified the input motion images of infants during GMs into Types 1–4 (*C* = 4), corresponding to WM, FM, CS, and PR, respectively. The entropy threshold used to prevent misclassifications caused by ambiguous input was set to ${S}_{{\rm{th}}}\,=\,1$. Thirty-one samples of each GM type were selected at random for leave-one-out cross-validation, and the classified results were tested by calculating the average of the classification results given by the PTs. The classification accuracies were averaged over five sets of cross-validation. The indices were standardized using the average values and standard deviations of normal GMs (i.e., WMs and FMs) used for the parameter adjustment of LLGMN (${N}_{c}\,=\,31$, total 62 samples).

The partial Kullback–Leibler (KL) information measure^26^ was used to reduce the number of indices and to evaluate the contribution of each index to the classification. Let *K*(*Q*, *C*) represent the KL information between the probabilistic distribution of input indices and that of classes, and $K(Q{\text{'}}_{[i]},C{\text{'}}_{[i]})$ be the KL information with the *i*-th input index reduced. The partial KL information is then defined by ${G}_{[i]}=K(Q{\text{'}}_{[i]},C{\text{'}}_{[i]})/K(Q,C)$. If the reduced *i*-th index does not contribute to the classification, the partial KL information becomes *G*_[*i*]_ = 1. By using the partial KL information, the input indices can be sequentially reduced to extract the most effective ones without evaluating all combinations of indices. In this experiment, 16 samples were randomly extracted for each type (total of 64 samples), and the classification accuracies were calculated with the input indices reduced one-by-one based on the partial KL information.

## Results

**Figure 4:**
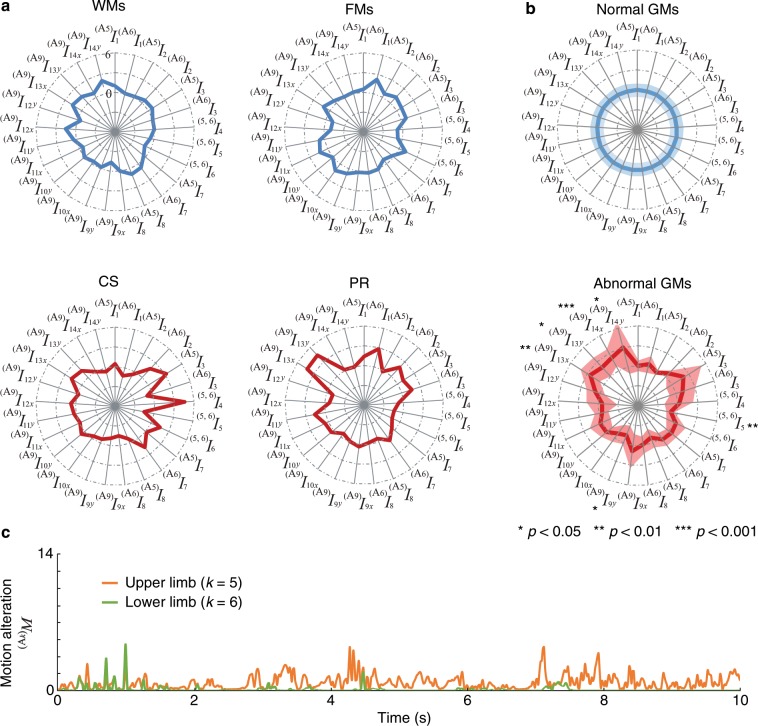
Results of motion analysis. (**a**) Examples of the radar charts of evaluation indices for each GM: WMs, FMs, CS, and PR. (**b**) Averaged radar charts of evaluation indices for normal and abnormal GMs. Solid lines and shaded areas represent the mean values and the standard deviations for all subjects, respectively. The statistical test results based on the unpaired two-tailed *t*-test are also shown. (**c**) Changes in movement ${}^{({A}_{5})}\,M$ and ${}^{({A}_{6})}\,M$ of CS.

Figure 4a shows examples of the radar charts produced by analysing the video images corresponding to each GM. The axes of these charts correspond to the input indices. To confirm the differences in the indices from the viewpoint of normality/abnormality of GMs, averaged radar charts for the normal GM group (WMs and FMs) and the abnormal GM group (CS and PR) were calculated for all subjects (Fig. 4b). The figure includes the statistical test results obtained by comparing the average value of each index of the normal GMs and the abnormal GMs based on the unpaired *t*-test (significance level: 5%). Statistically significant differences were observed in one of the indices of movement balance, ${}^{\mathrm{(5,6)}}\,{I}_{5}$, one of the indices of movement rhythm, ${}^{({\rm{A}}\mathrm{9)}}\,{I}_{9x}$, and the indices of movement of the body centre: ${}^{({\rm{A}}\mathrm{9)}}\,{I}_{13x}$, ${}^{({\rm{A}}\mathrm{9)}}\,{I}_{13y}$, ${}^{({\rm{A}}\mathrm{9)}}\,{I}_{14x}$, and ${}^{({\rm{A}}\mathrm{9)}}\,{I}_{14y}$. In addition, Fig. 4c shows the changes in body movement of the upper and lower limbs for the CS type.

**Figure 5:**
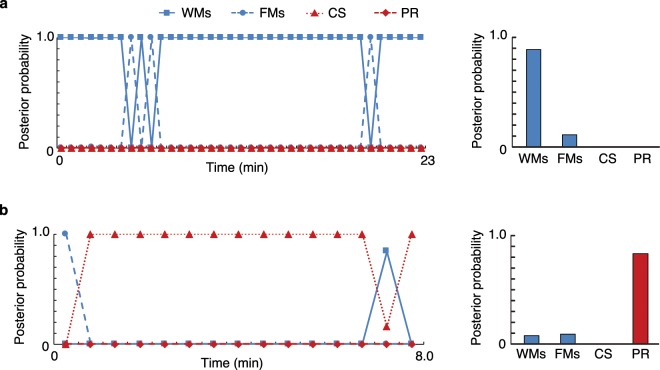
Time series and means of the posterior probabilities for each type. (**a**) Subject A. (**b**) Subject J. The results of the GM evaluator are “WMs” for subject A and “PR” for subject J. Note that each example has a different time scale on the horizontal axis, as the video length differs depending on the subjects (see Supplementary Table S1).

Figure 5 shows an example of the temporal change in the posterior probabilities of subjects A and J. The arithmetic mean of the posterior probabilities over all time intervals (except those used to learn the LLGMN parameters and those containing movements judged to be irrelevant by the PTs) are shown on the left-hand side of the figure. The motion with the highest posterior probability was Type 1 (WMs) for subject A and Type 4 (PR) for subject J.

**Figure 6:**
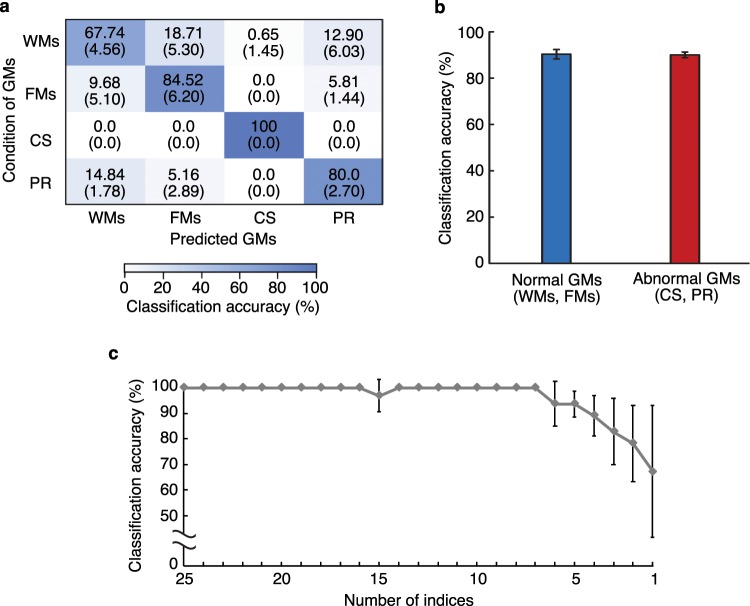
Classification results of the GMs. (**a**) Confusion matrix for the classification of four GM classifications. The rows and columns correspond to the PT assessments and the classification results of the system, respectively. Results averaged across five trials and standard deviation is shown. (**b**) Classification accuracy of normal GMs and abnormal GMs. Error bars represent standard deviations for all trials. (**c**) Classification accuracy of learning data for each number of indices. The input indices are reduced one-by-one using partial KL information. Error bars represent the standard deviations of the average classification accuracy of the respective GMs.

Figure 6a presents the average confusion matrix of the classification results across five trials. The rows and columns correspond to the PT assessments and the classification results of the system, respectively. The average precision^27^ of four classes over a total of five trials was 83.1 ± 1.84%. In addition, Fig. 6b shows the average successful classification accuracy of abnormal and normal motions. Here, Types 1 (WMs) and 2 (FMs) are defined as normal motion, and Types 3 (CS) and 4 (PR) are defined as abnormal motion based on the definition of GMs.

Figure 6c shows the average classification accuracy as the input indices are reduced one-by-one using partial KL information^26^. The average classification accuracies were calculated from the 64 samples used to calculate the partial KL information, and each error bar corresponds to the standard deviation of the average classification accuracy of the respective GMs.

## Discussion

Figure 4a shows that each index becomes approximately 0 for normal GMs (i.e., WMs and FMs). This is because the canonicalization of the indices was carried out using the normal GMs. In contrast, parts of the indices become large for the abnormal GMs (i.e., CS and PR), resulting in distorted radar charts. Specifically, the CS demonstrates that the ratio of the movement frequency between the upper and lower limbs ${}^{\mathrm{(5,6)}}\,{I}_{4}$ increases, indicating that the motion frequency of the lower body is much smaller than that of the upper body. As shown in Fig. 4c, the change in movement ${}^{({A}_{k})}\,M$ reinforces this fact, where the movement of the lower body is much smaller than that of the upper body in most time intervals. This can be caused by body rigidity, which is one of the characteristics of CS, in the lower body.

The differences between normal and abnormal GMs can also be confirmed from the averaged radar chart (Fig. 4b). For the indices relating to the movements of the body centre, the abnormal GMs showed a larger value than the normal GMs. This indicates that abnormal GMs usually involve quick and rough movements of the body centre (infants’ centre of mass estimated from images). Meanwhile, for the rhythm of the body centre and the balance of upper-limb and lower-limb, abnormal GMs showed smaller values than normal GMs. In general, short-lasting tremulous movement is a common feature of GM abnormalities^28^ and may lead to an increase in speed and fluctuation of the body centre and unbalanced non-rhythmical movement. Such movements are usually described as monotonic movements lacking fluency, and they are the main characteristics of abnormal GMs. These results confirm that the proposed system can effectively capture differences between normal and abnormal movements from the radar chart described by the proposed indices, and its findings are consistent with the definition of GMs.

Shown in Fig. 5, the assessment results given by the PTs indicate that typical and successive WMs were observed in subject A, and PR was continually observed in subject J. As the posterior probabilities calculated by the proposed system agree with these assessment results, the system is clearly useful as a diagnosis support tool.

The data presented in Fig. 6a, which shows that the average precision of four classes is 83.1 ± 1.84%, suggests that the proposed system is effective in accurately classifying four GM types. Figure 6b demonstrates that if the system is only required to distinguish between normal and abnormal motion, its performance improves to a classification accuracy of 90.2 ± 0.94%.

Figure 6c shows that the classification accuracy does not significantly decrease until the input indices have been reduced to six types. These six indices are the magnitudes of the fluctuation of the body centre ${}^{({A}_{9})}\,{I}_{{14}_{x}}$ and ${}^{({A}_{9})}\,{I}_{{14}_{y}}$, magnitude of the velocity of the body centre ${}^{({A}_{9})}\,{I}_{{14}_{x}}$, centre frequency of *G*^d^ ${}^{({A}_{k})}\,{I}_{{11}_{x}}$, movement count of the upper limbs ${}^{({A}_{5})}\,{I}_{3}$, and standard deviation of the change in movement of the upper limbs ${}^{({A}_{6})}\,{I}_{8}$. Among these six indices, four are related to the movement of the body centre, and the other two are related to the upper body. The following comments, partially consistent with the six indices, were given by the PTs regarding the infants participating in this experiment:

1. Subject J
  “The infant monotonously and repeatedly raised the right hand, and the left arm shows very little movement and is seemingly paralyzed”.
2. Subject B
  “Fidgety movement can be observed from both shoulders and both wrists”.
3. Subject C
  “A chain of movements, which is a feature of fidgety movement, was observed. For example, the movement started from the right arm, followed by the neck, left arm, left leg, body trunk, and, finally, right leg”.

Indices related to the upper body could be related to the first two comments, and those related to the body centre may be related to the third comment. Importantly, this analysis allowed us to identify effective indices to evaluate GMs.

In conclusion, for the early diagnosis of infant disorders, this paper has proposed a markerless movement measurement and evaluation system for GMs. The proposed system measures infant motion using a video camera, and it extracts motion indices based on clinical definitions through video image analysis. Based on the extracted indices, a probabilistic neural network is then used to classify motion types corresponding to GMs. Our experimental results show that the proposed system can capture the characteristics of the infants’ movements and accurately classify the motion types. Specifically, the classification accuracy of normal and abnormal motions reached 90.2 ± 0.94%, indicating that the proposed system may support early diagnoses.

We plan to test this system by performing long-term measurements in a neonatal intensive care unit (NICU) for infants with LBW who are considered to be at high risk of various disorders. To achieve this, it is necessary to automatically extract sections where the infant is performing GMs from the recorded video. Therefore, new algorithms for detecting sleep and crying intervals based on objective criteria should be introduced into the proposed system in the future. In addition, three-dimensional motion analysis may be required to increase the number of classifiable GMs and describe motions such as kicking that cannot be fully assessed by indices extracted from two-dimensional video images. To improve the classification accuracy, further analyses will include the definition of indices describing the motion characteristics in detail, such as those related to elegance of motion, stability of body centre, and temporal changes in GMs.

In this paper, the authors focused on automating the visual classification of GMs, whereas the proposed system can be applied not only to GM evaluation but also to a wide range of spontaneous movement analysis of infants. In the future, the authors would like to analyse infants with various risks (e.g. those who have experienced intraventricular haemorrhage), thereby allowing wider diagnosis support for PTs and doctors. This may require a detailed analysis between movement features evaluated via the proposed system and biological variables of infants. It is also important to evaluate the infant’s movements during sleep and crying, which were excluded from the analysis in this paper. Such segments other than GMs are known to have functional value in the neural development of infants^29,30^. Moreover, recent evidence suggests the importance of head movements in the study of atypical development^31^. However, the aim of this paper is to classify infant movement types based on Prechtl’s GM assessment, and the Prechtl method does not consider the head movements of infants. In the future, we plan to improve the system so that more detailed motion characteristics, including head movements, can be evaluated by introducing additional algorithms such as skeleton extraction or pose estimation.

## Appendix

### Calculation of the indices for movement analysis

#### Movement magnitude

The movement magnitude is evaluated by using the following three indices.

$${}^{({A}_{k})}\,{I}_{1}$$: Movement frequency

$${}^{({A}_{k})}\,{I}_{2}$$: Movement strength

$${}^{({A}_{k})}\,{I}_{3}$$: Movement count

First, movement occurrence is detected using a threshold value *M*_th_. The movement frequency ${}^{({A}_{k})}\,{I}_{1}$ is then defined as the ratio of frame numbers with movement detected to the total frame number *L* using the following equations:

9 $$\begin{array}{rcl}{}^{\left({A}_{k}\right)}\,{I}_{1} & = & \frac{100}{L}\mathop{\sum }\limits_{l\mathrm{=1}}^{L}\,{}^{\left({A}_{k}\right)}\,{\kappa }_{l},\\ {}^{\left({A}_{k}\right)}\,{\kappa }_{l} & = & \left\{\begin{array}{cc}1 & ({}^{\left({A}_{k}\right)}\,{M}_{l}\ge {M}_{{\rm{th}}})\\ 0 & ({}^{\left({A}_{k}\right)}\,{M}_{l} < {M}_{{\rm{th}}}).\end{array}\right.\end{array}$$

The movement strength ${}^{({A}_{k})}\,{I}_{2}$ is defined by the following equations:

10 $$\begin{array}{rcl}{}^{\left({A}_{k}\right)}\,{I}_{2} & = & \frac{1}{L^{\prime} }\mathop{\sum }\limits_{l\mathrm{=1}}^{L}\,{}^{\left({A}_{k}\right)}\,{\nu }_{l},\\ {}^{\left({A}_{k}\right)}\,{\nu }_{l} & = & \left\{\begin{array}{ll}{}^{\left(k\right)}\,{M}_{l} & ({}^{\left({A}_{k}\right)}\,{M}_{l}\ge {M}_{{\rm{th}}})\\ 0 & ({}^{\left({A}_{k}\right)}\,{M}_{l} < {M}_{{\rm{th}}}),\end{array}\right.\end{array}$$

where *L*′ represents the total frame number when ${}^{({A}_{k})}\,{M}_{l} > {M}_{{\rm{th}}}$. By defining ${}^{({A}_{k})}\,{l}_{q}^{{\rm{st}}}$ as the frame where ${}^{({A}_{k})}\,{M}_{l}\ge {M}_{{\rm{th}}}$, and ${}^{({A}_{k})}\,{l}_{q}^{{\rm{ed}}}$ as the successive frame where ${}^{({A}_{k})}\,{M}_{l}\le {M}_{{\rm{th}}}$, the interval $[{}^{({A}_{k})}\,{l}_{q}^{{\rm{st}}}\,{}^{({A}_{k})}\,{l}_{q}^{{\rm{ed}}}]$ is defined as the *q*-th occurrence of movement, enabling the movement count to be calculated using the following equation:

11 $${}^{({A}_{k})}\,{I}_{3}=\frac{Q}{L}.$$

Here, if ${}^{({A}_{k})}\,{l}_{Q}^{{\rm{ed}}}\ge L$, then ${}^{({A}_{k})}\,{l}_{Q}^{{\rm{ed}}}=L$.

#### Movement balance

The movement balance is evaluated by using the following three indices.

${}^{({k}_{1},{k}_{2})}I_{4}$: Ratio of index ${}^{({A}_{k})}\,{I}_{1}$ in upper and lower limbs

${}^{({k}_{1},{k}_{2})}I_{5}$: Ratio of index ${}^{({A}_{k})}\,{I}_{2}$ in upper and lower limbs

${}^{({k}_{1},{k}_{2})}I_{5}$: Symmetry in upper and lower limbs

Movement frequency ${}^{({A}_{k})}\,{I}_{1}$ and movement strength ${}^{({A}_{k})}\,{I}_{2}$ are used to calculate the ratio among different analysis areas and generate indices ${}^{({k}_{1},{k}_{2})}\,{I}_{4}$ and ${}^{({k}_{1},{k}_{2})}\,{I}_{5}$ by using the following equations:

12 $${}^{({k}_{1},{k}_{2})}\,{I}_{4}=\frac{{}^{({k}_{1})}\,{I}_{1}}{{}^{({k}_{2})}I_{1}}$$

13 $${}^{({k}_{1},{k}_{2})}\,{I}_{5}=\frac{{}^{({k}_{1})}\,{I}_{1}}{{}^{({k}_{2})}I_{1}}$$

where *k*_1_, *k*_2_ (${k}_{1},\,{k}_{2}\,=\,\mathrm{1,}\,\mathrm{2,}\,\cdots ,\,8$) represent analysis areas other than the whole-body area, and ${k}_{1}\ne {k}_{2}$. Note that if ${}^{({k}_{2})}\,{I}_{1}=0$, ${}^{({k}_{2})}\,{I}_{2}=0$, then ${}^{({k}_{1},{k}_{2})}\,{I}_{4}$ = 0, ${}^{({k}_{1},{k}_{2})}\,{I}_{5}=0$. ${}^{({k}_{1},{k}_{2})}\,{I}_{6}$ is defined as the normalized cross-correlation between ${}^{({k}_{1})}\,{M}_{l}$ and ${}^{({k}_{2})}\,{M}_{l}$ in different analysis areas. This is calculated by the following equations:

14 $${}^{({k}_{1},{k}_{2})}\,{I}_{6}=\frac{\mathop{\sum }\limits_{l\mathrm{=1}}^{L}\,({}^{({k}_{1})}\,{M}_{l}-{}^{({k}_{1})}\,{M}^{{\rm{ave}}.})({}^{({k}_{2})}\,{M}_{l}-{}^{(k\mathrm{2)}}\,{M}^{{\rm{ave}}.})}{\,\sqrt{\mathop{\sum }\limits_{l\mathrm{=1}}^{L}\,{({}^{({k}_{1})}M{}_{l}-{}^{({k}_{1})}{M}^{{\rm{ave}}.})}^{2}}\,\sqrt{\mathop{\sum }\limits_{l\mathrm{=1}}^{L}\,{({}^{({k}_{2})}M{}_{l}-{}^{({k}_{2})}{M}^{{\rm{ave}}.})}^{2}}},$$

where ${}^{({k}_{1})}\,{M}^{{\rm{ave}}.}$ and ${}^{({k}_{2})}\,{M}^{{\rm{ave}}.}$ represent the average value of ${}^{({k}_{1})}\,{M}_{l}$ and ${}^{({k}_{2})}\,{M}_{l}$ over the total frame number *L*, respectively.

#### Movement rhythm

The movement rhythm includes the following six indices.

${}^{({A}_{k})}\,{I}_{7}$: Centre frequency of ${}^{({A}_{k})}\,{M}_{l}$

${}^{({A}_{k})}\,{I}_{8}$: Second moment around centre frequency ${}^{({A}_{k})}\,{I}_{7}$

${}^{({A}_{9})}\,{I}_{{9}_{x}},{}^{({A}_{9})}\,{I}_{{9}_{y}}$: Centre frequency of *G*^v^

${}^{({A}_{9})}\,{I}_{{10}_{x}},{}^{({A}_{9})}\,{I}_{{10}_{y}}$: Second moment around the centre frequencies of ${}^{({A}_{9})}\,{I}_{{9}_{x}}$ and ${}^{({A}_{9})}\,{I}_{{9}_{y}}$

${}^{({A}_{9})}\,{I}_{{11}_{x}},{}^{({A}_{9})}\,{I}_{{11}_{y}}$: Centre frequency of *G*^*d*^

${}^{({A}_{9})}\,{I}_{{12}_{x}},{}^{({A}_{9})}\,{I}_{{12}_{y}}$: Second moment around the centre frequencies of ${}^{({A}_{9})}\,{I}_{{11}_{x}}$ and ${}^{({A}_{9})}\,{I}_{{11}_{y}}$

To calculate the above indices, a fast Fourier transform is applied to the time series data of the changes in body movement ${}^{({A}_{k})}\,{M}_{l}$, velocity of the body centre (${G}_{l,x}^{{\rm{v}}}$, ${G}_{l,y}^{{\rm{v}}}$), and fluctuation of the body centre (${G}_{l,x}^{{\rm{d}}}$, ${G}_{l,y}^{{\rm{d}}}$). The resulting power spectrum density (PSD) distributions are then normalized according to their maximum values, and the centre frequencies and second moments around the centre frequencies are calculated using the following equations:

15 $${F}_{{\rm{cntr}}.}=\frac{\mathop{\sum }\limits_{f\mathrm{=0}}^{{f}_{{\rm{\max }}}}\,fP(f)}{\mathop{\sum }\limits_{f\mathrm{=0}}^{{f}_{{\rm{\max }}}}\,P(f)},$$

16 $${D}_{{\rm{cntr}}.}=\sqrt{\frac{\mathop{\sum }\limits_{f\mathrm{=0}}^{{f}_{{\rm{\max }}}}\,P(f)(f-{F}_{{\rm{cntr}}.}{)}^{2}}{\mathop{\sum }\limits_{f\mathrm{=0}}^{{f}_{{\rm{\max }}}}\,P(f)}},$$

where *P*(*f*) represents the normalized PSD and *f*_max_ is the maximum frequency range for analysis. The centre frequencies are then calculated for each analysis interval *L*_f_ (${L}_{{\rm{f}}}\le L$, $\frac{L}{{L}_{{\rm{f}}}}\in {\mathbb{N}}$) as well as the average values of the reciprocals of the second moment around the centre frequencies.

#### Movement of the body centre

The movement of the body centre is evaluated using the following indices.

${}^{({A}_{9})}\,{I}_{{13}_{x}},\,{}^{({A}_{9})}\,{I}_{{13}_{y}}$: Magnitude of the velocity of the body centre

${}^{({A}_{9})}\,{I}_{{14}_{x}},\,{}^{({A}_{9})}\,{I}_{{14}_{y}}$: Magnitude of the fluctuation of the body centre

These indices are defined as the average value over the total frame number *L* of the velocity of the body centre (${G}_{l,x}^{{\rm{v}}}$, ${G}_{l,y}^{{\rm{v}}}$) and the change in the body centre (${G}_{l,x}^{{\rm{d}}}$, ${G}_{l,y}^{{\rm{d}}}$), respectively.

17 $$\left({}^{\left({A}_{9}\right)}\,{I}_{{13}_{x}},\,{}^{\left({A}_{9}\right)}\,{I}_{{13}_{y}}\right)=\left(\frac{1}{L}\mathop{\sum }\limits_{l\mathrm{=1}}^{L}{G}_{l,x}^{{\rm{v}}}\,,\,\frac{1}{L}\mathop{\sum }\limits_{l\mathrm{=1}}^{L}{G}_{l,y}^{{\rm{v}}}\right),$$

18 $$\left({}^{\left({A}_{9}\right)}\,{I}_{{14}_{x}},\,{}^{\left({A}_{9}\right)}\,{I}_{{14}_{y}}\right)=\left(\frac{1}{L}\mathop{\sum }\limits_{l\mathrm{=1}}^{L}\,{G}_{l,x}^{{\rm{d}}},\,\frac{1}{L}\mathop{\sum }\limits_{l\mathrm{=1}}^{L}\,{G}_{l,y}^{{\rm{d}}}\right).$$

## Supplementary information

Supplementary Information.

## Data Availability

The datasets generated and/or analysed in the current study are available from the corresponding author upon reasonable request.
