# Supplementary material for: Markerless Measurement and Evaluation of General Movements in Infants
Source: Sci Rep. 2020 Jan 29;10:1422. doi: 10.1038/s41598-020-57580-z (PMC6989465; doi:10.1038/s41598-020-57580-z)
Supplement: Supplementary file 1 — Supplementary Information. [file 41598_2020_57580_MOESM1_ESM.pdf]

Supplementary material

Markerless Measurement and Evaluation of General Movements in Infants

Toshio Tsuji[1*], Shota Nakashima[1], Hideaki Hayashi[2], Zu Soh[1], Akira Furui[1], Taro Shibanoki[3], Keisuke Shima[4], and Koji Shimatani[5]

1 Department of System Cybernetics, Graduate School of Engineering, Hiroshima University, 1-4-1 Kagamiyama, Higashi-Hiroshima, Hiroshima 739-8527, Japan

2 Faculty of Information Science and Electrical Engineering, Kyushu University, 744 Motooka, Nishi-Ku, Fukuoka, Fukuoka 819-0395, Japan

3 College of Engineering, Ibaraki University, 4-12-1 Nakanarusawa, Hitachi, Ibaraki 316-8511, Japan

4 School of Engineering Science, Yokohama National University, 79-1 Tokiwadai, Hodogaya Ward, Yokohama, Kanagawa 240-8501, Japan

5 Department of Physical Therapy, Prefectural University of Hiroshima, 1-1 Gakuen, Mihara, Hiroshima 723-0053, Japan

The PDF file includes:

Supplementary Table S1. Subject information

Supplementary Table S1. Subject information. For subjects with multiple videos, the average video lengths (and standard deviations) are shown.

Subject	Type of GMs	Gestational age (week)	Corrected age (week)	Birth weight (g)	Gender	Number of videos	Video length (s)	Number of using analysis intervals	Place of recording
A	WMs, FMs	-	2–8 (WMs)<br>9–15 (FMs)	3,175	Male	9	640±346.6	37 (WMs)<br>155 (FMs)	Home
B	WMs, FMs	-	3–8 (WMs)<br>8–15 (FMs)	3,068	Female	6	125±35.1	11 (WMs)<br>14 (FMs)	Home
C	WMs, FMs	-	2–7 (WMs)<br>8–15 (FMs)	2,960	Female	14	357.9±227.4	57 (WMs)<br>110 (FMs)	Home
D	WMs	37	-	2,246	Female	1	540	9	Hospital
E	WMs	29	-	1,024	Female	1	600	14	Hospital
F	WMs	32	-	1,184	Male	1	570	9	Hospital
G	WMs	34	-	1,648	Male	1	600	18	Hospital
H	WMs	35	-	2,240	Male	1	510	7	Hospital
I	WMs	35	-	2,184	Female	1	1,170	17	Hospital
J	PR	-	-	-	-	1	480	15	Hospital
K	PR	-	-	-	-	1	600	20	Hospital
L	PR	-	-	-	-	1	660	22	Hospital
M	WMs	-	-	-	-	1	420	14	Hospital
N	PR	-	-	-	-	1	570	7	Hospital
O	PR	-	-	-	-	1	540	10	Hospital
P	PR	-	-	-	-	1	510	13	Hospital
Q	FMs	-	-	-	-	1	450	12	Hospital
R	PR	-	-	-	-	1	540	18	Hospital
S	PR	-	-	-	-	1	390	4	Hospital
T	CS	28	-	1,200	Male	1	60	31 †	Hospital
U	PR	25	-	860	Female	1	60	31 †	Hospital

† The overlapped interval was used for subject T and subject U because their videos were shorter than those of the other
